# Potential Physiological Convergence Between GLP-1 Receptor Signaling and Hepatocyte TAZ in Metabolic Liver Disease: From Metabolic Homeostasis to Fibrotic Remodeling

**DOI:** 10.3390/cells15191813

**Published:** 2026-10-04

**Authors:** Mohammed F. Gholam

**Affiliations:** 1 King Abdullah International Medical Research Center, Jeddah 21423, Saudi Arabia; gholamm@ksau-hs.edu.sa; 2 Department of Basic Medical Sciences, College of Medicine, King Saud bin Abdulaziz University for Health Sciences, Jeddah 21423, Saudi Arabia; 3 Ministry of the National Guard–Health Affairs, Jeddah 21423, Saudi Arabia

**Keywords:** glucagon-like peptide-1, TAZ, WWTR1, MASH, hepatic fibrosis, mechanotransduction

## Abstract

**Highlights:**

**What are the main findings?**

- GLP-1 receptor agonists reduce metabolic and inflammatory inputs that promote steatohepatitis, whereas hepatocyte TAZ independently integrates lipid, mechanical, and profibrotic stress.
- Mouse studies identify liver sinusoidal endothelial cells as functionally relevant intrahepatic GLP-1R-expressing cells, but a downstream effect on hepatocyte TAZ has not been demonstrated.

**What are the implications of the main findings?**

- The evidence supports systemic metabolic unloading and an endothelial-mediated intrahepatic pathway; direct hepatocyte GLP-1R-to-TAZ signaling remains hypothetical.
- Future studies should combine cell-specific receptor perturbation, weight-matched controls, spatial liver models, and direct TAZ-TEAD activity measurements.

**Abstract:**

Metabolic dysfunction-associated steatotic liver disease (MASLD) progresses when nutrient excess, insulin resistance, adipose dysfunction, and hepatocellular stress overwhelm hepatic adaptation. Glucagon-like peptide-1 receptor agonists (GLP-1RAs) improve weight, glycemic control, and steatohepatitis, and semaglutide has demonstrated histological improvement in metabolic dysfunction-associated steatohepatitis (MASH). In parallel, the transcriptional coactivator with PDZ-binding motif (TAZ; WWTR1) has emerged as a nutrient-, cholesterol-, and mechanics-responsive regulator linking stressed hepatocytes to inflammation and fibrogenesis through Hippo-YAP/TAZ signaling and Indian hedgehog. This narrative review integrates incretin physiology with cell-specific TAZ biology across the gut–brain–axis, adipose tissue, pancreatic islets, liver sinusoidal endothelial cells, hepatocytes, immune cells, and hepatic stellate cells. Recent evidence that semaglutide can signal through intrahepatic sinusoidal endothelial GLP-1 receptors provides a weight-loss-independent route to reduced hepatic inflammation and fibrosis; however, it does not establish that TAZ is a downstream mediator. The evidence therefore supports a multilevel convergence model in which GLP-1RAs lower metabolic and inflammatory inputs that favor TAZ activation, while direct GLP-1RA-to-TAZ signaling remains a testable hypothesis. Receptor-validated, cell-specific, weight-controlled, and longitudinal human studies are needed to distinguish mediation from parallel response and to determine whether TAZ-pathway biomarkers can guide mechanism-based therapy.

## 1. Introduction

Metabolic dysfunction-associated steatotic liver disease is the hepatic expression of a multisystem disturbance involving energy balance, adipose storage capacity, insulin action, inflammation, and vascular-metabolic risk [1]. Its progressive inflammatory phenotype, MASH, is clinically important because fibrosis stage predicts liver-related outcomes more strongly than steatosis alone [1]. The key physiological question is not simply why triglyceride accumulates in hepatocytestriglycerides accumulate, but why some steatotic livers move from adaptive lipid storage to hepatocyte injury, inflammatory signaling, extracellular-matrix deposition, and architectural distortion [1].

GLP-1RAs reproduce or amplify an endogenous incretin signal that coordinates nutrient intake with insulin secretion, glucagon suppression, gastric emptying, satiety, and body-weight regulation [2,3,4]. Their actions reduce several upstream drivers of hepatic injury [2,3,4]. Histology-based trials have moved this drug class from a plausible metabolic adjunct toward a disease-modifying strategy in MASH [5,6]. Yet the cellular pathway connecting whole-body metabolic improvement to regression of hepatic inflammation and fibrosis remains incompletely defined [3,7,8].

TAZ and its paralogue YAP are transcriptional coactivators regulated by the Hippo kinase cascade, cytoskeletal tension, cell polarity, matrix stiffness, G-protein signaling, and metabolic inputs [9,10,11]. In hepatocytes, TAZ rises during steatohepatitis and can induce paracrine mediators that activate hepatic stellate cells [12]. TAZ may therefore be more than a nonspecific fibrosis marker; it may help drive the transition from metabolic stress to maladaptive tissue repair [10,11,12]. This review integrates GLP-1 physiology, hepatic intercellular communication, and TAZ mechanotransduction; critically evaluates the evidence for convergence; and defines experiments capable of separating direct receptor signaling from secondary effects of weight loss and improved metabolic homeostasis.

Existing reviews have separately summarized the metabolic and clinical effects of GLP-1-based therapies and the roles of Hippo-YAP/TAZ signaling in liver injury. The specific contribution of this review is to integrate those fields using a cell-specific hierarchy of evidence: established systemic physiology, genetically supported intrahepatic GLP-1 receptor signaling in experimental MASH, and still-hypothetical connections to hepatocyte TAZ. The review therefore emphasizes falsifiable predictions and experimental designs rather than presenting a GLP-1R-TAZ pathway as established.

## 2. Methods: Literature Identification and Synthesis

An updated targeted narrative search was conducted in PubMed from database inception through 29 September 2026. Search combinations included glucagon-like peptide-1, GLP-1 receptor agonist, semaglutide, liraglutide, tirzepatide, MASLD, MASH, NASH, hepatic fibrosis, Hippo, YAP, TAZ, WWTR1, TEAD, Indian hedgehog, liver sinusoidal endothelial cell, hepatic stellate cell, hepatocyte, receptor localization, single-cell, cell-resolved, and genetic deletion. Reference lists of relevant primary studies and recent reviews were also examined. Titles and abstracts were screened for mechanistic or clinical relevance, and full texts were prioritized when they provided receptor-validated or cell-specific evidence, genetic causality, human tissue data, histological trial outcomes, or direct TAZ-pathway measurements. Evidence was organized by physiological level and by strength of inference. This was a narrative rather than systematic review; no formal protocol, duplicate independent screening, quantitative risk-of-bias assessment, or meta-analysis was performed.

## 3. GLP-1 as an Integrative Physiological Signal

### 3.1. Endogenous Incretin Physiology

GLP-1 is secreted predominantly from intestinal enteroendocrine L cells after nutrient exposure, although neural and paracrine mechanisms contribute to the rapid postprandial response [2,3,4]. Binding of GLP-1 to its class B G-protein-coupled receptor increases cyclic adenosine monophosphate in pancreatic beta cells and potentiates insulin secretion in a glucose-dependent manner [2,3,4]. GLP-1 also suppresses glucagon when glucose is elevated, slows gastric emptying, promotes satiety through gut–brain– and central circuits, and modifies reward-related feeding behavior [2,3,4]. The combined effect is a coordinated reduction in postprandial glucose excursions and energy intake without obligate insulin secretion during hypoglycemia [2,3,4].

For the liver, many GLP-1RA effects are physiologically indirect [2,3]. Reduced caloric intake and weight loss decrease adipose-tissue mass and lipolytic fatty-acid delivery [2,3]. Improved insulin action suppresses inappropriate hepatic glucose production and decreases de novo lipogenesis [2,3]. Lower glucagon action can reduce hepatic substrate flux, whereas improved adipose storage function and reduced inflammatory signaling lower the exposure of hepatocytes to fatty acids, ceramides, cytokines, and other stressors [2,3]. These systemic effects are sufficiently powerful to change liver biology even if hepatocytes express little or no functional canonical GLP-1 receptor [3,7,13].

### 3.2. Temporal and Dose-Dependent Features of Incretin Physiology

The physiological response to GLP-1 receptor agonism unfolds across different time scales [2,3,4]. Gastric emptying and postprandial glucose handling may change soon after treatment begins, whereas sustained reductions in energy intake, adipose mass, insulin resistance, and hepatic lipid burden develop over weeks to months [2,3,4]. This temporal separation matters when interpreting liver outcomes. An early fall in aminotransferases or hepatic fat may reflect altered substrate delivery and improved metabolic flux [5,6], while measurable regression of collagen-rich matrix generally requires longer exposure and coordinated changes in injury, inflammation, matrix synthesis, and matrix degradation [1,14,15].

Pharmacological agents within the incretin class differ in receptor selectivity, exposure, dosing interval, central effects, gastrointestinal tolerability, and magnitude of weight loss [2,3]. Semaglutide provides selective GLP-1 receptor agonism, whereas tirzepatide combines GIP and GLP-1 receptor activity [3,5,16]. Similar histological outcomes therefore do not necessarily imply identical intracellular pathways [5,16]. Mechanistic studies should record achieved drug exposure, weight trajectory, food intake, glycemic change, and body-composition change rather than treating treatment assignment alone as the biological dose.

Interindividual heterogeneity is equally important. Baseline obesity, diabetes duration, adipose-tissue dysfunction, fibrosis stage, dietary composition, genetic susceptibility, and concomitant therapies can alter both the metabolic response to treatment and the hepatic stress landscape. A patient with marked adipose lipolysis and insulin resistance may improve mainly through reduced fatty-acid flux, whereas another patient with advanced matrix remodeling may require prolonged treatment before stiffness-dependent signaling changes. This heterogeneity provides a physiological rationale for pairing conventional clinical endpoints with pathway-resolved biomarkers and for avoiding a single-mechanism interpretation of GLP-1RA benefit.

### 3.3. The Unresolved Question of Direct Hepatic GLP-1 Receptor Signaling

Whether GLP-1RAs act directly on hepatocytes or hepatic stellate cells remains controversial [7,8,13,17]. Reports of hepatic GLP-1 receptor expression vary with species, disease state, assay specificity, and cell preparation [7,8,13,17]. Receptor antibodies and low-abundance transcripts require particular caution [7,13]. Consequently, improvements in steatosis, inflammation, or fibrosis should not automatically be attributed to a direct hepatocyte GLP-1 receptor pathway [7,8,13]. A rigorous physiological framework separates receptor-proven cell-autonomous effects from secondary effects mediated by weight loss, glycemic control, reduced adipose lipolysis, altered gut–liver– signaling, immune modulation, and changes in hepatic perfusion or substrate delivery [2,3,8].

Earlier reports supported direct hepatocyte signaling. In primary human hepatocytes and hepatoma-derived cell lines, GLP-1 receptor immunoreactivity and transcript detection were accompanied by ligand internalization, activation of insulin-signaling intermediates, and reduced triglyceride accumulation after GLP-1 or exendin-4 exposure [17]. These findings established biological plausibility but remain difficult to generalize because transformed cell lines can acquire nonphysiological receptor profiles, primary hepatocytes dedifferentiate in culture, and several historically used antibodies lacked adequate specificity.

More stringent localization studies reached a different conclusion. An extensively validated monoclonal antibody localized human and nonhuman-primate GLP-1 receptors to canonical pancreatic and selected extra-pancreatic targets but did not establish convincing hepatocyte expression [13]. More recently, receptor profiling and functional experiments in human hepatocytes and hepatic stellate cells found no meaningful direct response to GLP-1 or GIP receptor agonism [7]. Collectively, these studies argue against treating hepatocytes or stellate cells as established direct pharmacological targets and emphasize the need for genetic receptor dependence rather than antibody staining alone.

### 3.4. Liver Sinusoidal Endothelial Cells Revise the Direct-Liver Model

A 2026 study substantially refined this debate by identifying functional intrahepatic GLP-1 receptors in liver sinusoidal endothelial cells and selected immune-cell populations rather than in hepatocytes. In mouse models of metabolic liver disease, semaglutide improved steatosis, inflammation, and fibrosis even when weight loss was experimentally dissociated from treatment [18]. Cell-specific receptor deletion and transcriptional profiling implicated sinusoidal endothelial signaling in these weight-loss-independent benefits [8]. This observation provides a credible direct hepatic route while preserving the principle of cell specificity.

The endothelial finding is particularly relevant to TAZ biology because sinusoidal endothelial cells regulate immune-cell recruitment, vascular tone, paracrine communication, and the stellate-cell niche. Reduced endothelial inflammatory signaling could diminish hepatocyte stress and matrix remodeling, thereby lowering mechanical and metabolic inputs that stabilize TAZ. Nevertheless, no study has yet shown that endothelial GLP-1 receptor signaling changes hepatocyte TAZ abundance, nuclear localization, TEAD occupancy, or IHH secretion. The most defensible interpretation is therefore a cell-to-cell convergence model, not a demonstrated linear receptor pathway.

Table 1 summarizes the cell-specific evidence that defines the current direct-liver model. The strongest functional evidence supports an endothelial mechanism in mouse MASH, whereas hepatocyte and stellate-cell GLP-1 receptor signaling remains unestablished.

## 4. From Metabolic Overload to Fibrotic Remodeling

Hepatic triglyceride storage is initially an adaptive response that buffers potentially toxic fatty acids [1]. Injury emerges when lipid influx, synthesis, oxidation, and export become imbalanced and when toxic lipid species, free cholesterol, oxidative stress, endoplasmic-reticulum stress, mitochondrial dysfunction, and impaired autophagy disrupt cellular homeostasis [1,10]. Damaged hepatocytes release cytokines, chemokines, extracellular vesicles, damage-associated molecular patterns, and developmental morphogens [10]. Kupffer cells and recruited monocyte-derived macrophages amplify inflammation, while sinusoidal endothelial dysfunction alters vascular tone, permeability, and paracrine support [1,10].

Hepatic stellate cells are normally perisinusoidal, vitamin-A-storing cells that help maintain matrix homeostasis [14]. Persistent injury transforms them into proliferative, contractile myofibroblast-like cells that express alpha-smooth muscle actin and synthesize collagen-rich extracellular matrix [14]. The matrix is not merely the endpoint of injury. Increasing stiffness changes integrin signaling, actomyosin tension, nuclear architecture, and transcription, thereby reinforcing YAP/TAZ activity and fibrogenic behavior [9,15]. Fibrosis is thus a self-reinforcing physiological state in which biochemical and mechanical signals converge [9,14,15].

### The Sinusoidal Unit as an Integrated Injury-Repair System

The liver sinusoid should be considered a functional unit rather than a collection of isolated cell types [8,14]. Hepatocytes receive nutrients and hormones from portal blood; liver sinusoidal endothelial cells regulate exchange, vascular tone, leukocyte adhesion, and paracrine support; Kupffer cells sense microbial and damage-associated signals; and stellate cells respond to inflammatory, developmental, and mechanical cues [8,14]. Because these cells share a confined microenvironment, a primary perturbation in one compartment can be amplified through several others [8,14]. This organization explains how an endothelial GLP-1 receptor signal could modify hepatocyte stress and fibrogenesis without requiring direct receptor expression on hepatocytes or stellate cells [8].

Disease progression also involves changes in zonation. Pericentral and periportal hepatocytes differ in oxygen exposure, nutrient metabolism, and susceptibility to toxic or lipogenic stress, while endothelial phenotypes vary along the sinusoidal axis. The reported enrichment of Glp1r in pericentral LSECs in experimental MASH is therefore physiologically meaningful because this region overlaps with metabolic and hypoxic stresses that commonly accompany steatohepatitis [8]. Spatial localization should be retained in future analyses; averaging receptor abundance or TAZ activity across whole-liver tissue can erase a biologically important zonal response.

Fibrosis progression and regression are both active processes [14,15]. Activated stellate cells produce extracellular matrix, but macrophage phenotype, matrix metalloproteinase activity, tissue inhibitors of metalloproteinases, endothelial recovery, and removal of the injurious stimulus influence whether scar is maintained or remodeled [14,15]. Accordingly, reduced collagen deposition after incretin therapy could result from lower hepatocyte injury, diminished stellate-cell activation, enhanced matrix degradation, or a combination of these processes. TAZ may participate in the injury-to-activation phase, but histological fibrosis improvement alone cannot specify which component of matrix turnover changed [14,15].

## 5. TAZ Biology: A Sensor of Metabolism, Mechanics, and Injury

TAZ is encoded by WWTR1 and functions with TEAD-family DNA-binding factors to regulate gene programs involved in survival, proliferation, differentiation, inflammation, and matrix remodeling [9,11,15]. Under active canonical Hippo signaling, MST1/2-associated kinases activate LATS1/2, which phosphorylate TAZ and YAP, promoting cytoplasmic retention and proteasomal degradation [9,11,15]. When Hippo restraint is reduced, or when actin polymerization and mechanical tension favor nuclear entry, TAZ accumulates in the nucleus and coactivates context-dependent transcription [9,11,15].

Through this regulatory architecture, TAZ can translate the physical and metabolic state of a tissue into changes in gene expression [9]. Cell density, polarity, adhesion, G-protein-coupled receptor signaling, extracellular-matrix stiffness, Rho-family GTPases, and cellular energy status all influence YAP/TAZ [9]. In liver physiology, tightly regulated YAP/TAZ activity contributes to development, regeneration, bile-duct biology, and maintenance of tissue architecture [10,11]. Persistent or cell-inappropriate activation, however, promotes maladaptive regeneration, inflammation, fibrogenesis, and tumorigenesis [10,11].

### 5.1. Hepatocyte TAZ as a Driver of Steatohepatitis-Associated Fibrosis

The most direct mechanistic evidence is TAZ-specific and comes from hepatocyte-focused studies. TAZ abundance and nuclear activity are increased in hepatocytes from human and murine steatohepatitis. Hepatocyte-directed TAZ silencing can prevent or reverse inflammation, cell injury, and fibrosis in dietary mouse models without necessarily eliminating steatosis, indicating that TAZ participates in the transition from lipid storage to inflammatory fibrogenesis. A principal TAZ-specific mechanism is TAZ-TEAD-dependent induction of Indian hedgehog, which is secreted by hepatocytes and stimulates fibrogenic programs in stellate cells [12]. Other mechanotransduction studies cited in this review evaluate YAP and TAZ together and should not be interpreted as proof of a TAZ-specific function [9,15,19].

Nutrient composition also matters. Free cholesterol can stabilize hepatocyte TAZ through an ASTER-soluble adenylyl cyclase-calcium-RhoA pathway that limits beta-TrCP-mediated TAZ degradation [20]. This finding connects lipid handling to mechanotransduction rather than treating them as separate disease domains [20].

### 5.2. Cell Specificity and Conflicting Evidence

TAZ biology is highly cell- and context-dependent. Hepatocyte TAZ appears profibrogenic in several MASH models, but deletion of YAP/TAZ in myofibroblasts has been reported to be dispensable for murine liver fibrosis [19]. This apparent conflict is instructive: pathway importance cannot be inferred from whole-liver expression or generalized from one mesenchymal population to another. Timing, injury model, lineage definition, compensatory signaling, and YAP versus TAZ dependence may alter results. A publishable model must therefore specify the cell type, disease stage, upstream stimulus, and readout [12,19]. The multilevel physiological relationships are summarized in Table 2.

### 5.3. TAZ as a Dynamic Mechanosensor and Feedback Amplifier

TAZ should not be understood as a binary fibrosis switch. Its nuclear activity reflects the balance between Hippo-kinase-dependent phosphorylation and a wider set of mechanical and metabolic inputs [9,10,11,15]. Cell polarity, junctional integrity, actomyosin tension, integrin engagement, Rho-family GTPases, and extracellular-matrix stiffness all influence the nuclear availability of YAP and TAZ [9,15]. In a healthy liver, this responsiveness supports development, regeneration, and adaptation to changing metabolic demand [10,11]. During persistent injury, the same system can convert altered tissue architecture into sustained transcriptional activity [10,11,15].

This framework is important for interpreting therapeutic response. A drug does not need to bind TAZ or TEAD directly to change pathway activity. Any intervention that reduces hepatocyte swelling, oxidative stress, cytoskeletal tension, inflammatory signaling, or matrix stiffness may alter the probability that TAZ enters the nucleus and remains transcriptionally active. Conversely, advanced cross-linked fibrosis may preserve mechanical activation even after steatosis and inflammation improve. This may help explain why resolution of steatohepatitis can precede measurable regression of established fibrosis.

A further complication is the partial overlap and non-equivalence of YAP and TAZ. Many antibodies, gene signatures, and experimental interventions assess the two coactivators together, yet hepatocyte TAZ has been linked particularly strongly to IHH-dependent fibrogenic communication in dietary steatohepatitis [12,20,21,22,23,24]. Studies of GLP-1RA treatment should therefore report TAZ and YAP separately, distinguish total from phosphorylated and nuclear protein, and measure TEAD-dependent transcription rather than relying on whole-liver abundance. Spatial methods are needed because a fall in hepatocyte TAZ could be obscured by pathway activity in ductular, endothelial, immune, or stromal compartments.

### 5.4. Interactions with Metabolic and Inflammatory Signaling

TAZ does not operate in isolation from canonical metabolic signaling. Insulin resistance, excess fatty-acid delivery, oxidative stress, endoplasmic-reticulum stress, and mitochondrial dysfunction can alter cytoskeletal organization and survival signaling, thereby changing the context in which Hippo kinases regulate TAZ [10,11,12]. Conversely, TAZ-dependent transcription can reshape the cellular secretome and influence neighboring immune and mesenchymal cells [12,21]. This bidirectionality makes TAZ a plausible integrator of metabolic and mechanical stress, but it also means that changes in TAZ may be both a cause and a consequence of improving tissue physiology [10,11,12,21].

The hepatocyte TAZ-IHH pathway provides the clearest disease-specific example. In dietary models, increased hepatocyte TAZ promotes IHH expression and paracrine activation of fibrogenic programs, while hepatocyte-directed TAZ silencing reduces inflammation and fibrosis [12,22,23]. The humanized-liver study extends this concept by showing that silencing TAZ in human hepatocytes within a chimeric mouse can reduce profibrogenic signaling and fibrosis [22]. Even so, these interventions do not establish that GLP-1 receptor agonism uses the same pathway. Demonstrating convergence requires showing that incretin treatment changes hepatocyte TAZ activity and that preventing this change modifies the hepatic treatment response.

Mechanistic readouts must therefore go beyond WWTR1 messenger RNA or total TAZ protein. Nuclear localization, phosphorylation state, TEAD occupancy, and downstream transcription provide more direct evidence of pathway activity. IHH is relevant because it links hepatocyte TAZ to intercellular fibrogenic signaling, whereas broader YAP/TAZ targets such as CTGF and CYR61 may arise from several hepatic cell populations. Ideally, spatial transcriptomics or multiplex imaging should identify the cell producing each signal and relate it to local matrix composition, endothelial phenotype, and stellate-cell activation [12,21,22,24].

## 6. A Physiological Convergence Model for GLP-1RAs and TAZ

Taken together, the evidence suggests several points of convergence, but it does not establish a single linear pathway. Four routes merit consideration. First, weight-loss-dependent unloading reduces adipose lipolysis, hepatic fatty-acid delivery, de novo lipogenesis, and free-cholesterol stress; this may lessen the signals that stabilize or activate hepatocyte TAZ [3,5,6,16,20]. Second, weight-loss-independent metabolic effects may improve insulin action, inflammatory tone, endothelial function, and substrate partitioning before substantial weight change [3,8]. Third, reduced hepatocyte injury may decrease TAZ-dependent paracrine communication with stellate cells and macrophages [12,21,22,23]. Fourth, gradual reduction in matrix deposition and tissue stiffness may diminish mechanosensitive nuclear YAP/TAZ signaling, forming a beneficial reverse-feedback loop [9,15].

A direct receptor pathway remains possible, but it should be regarded as a hypothesis until functional GLP-1 receptor expression is demonstrated in the relevant human liver cell and receptor dependence is confirmed with genetic or pharmacological controls [3,7,8,13,17]. If present, canonical Gs-cAMP-protein kinase A signaling could influence RhoA, cytoskeletal tension, kinase activity, or transcriptional partners upstream of TAZ [9]. However, the same downstream changes could arise indirectly from systemic metabolic improvement [2,3]. Experimental designs must therefore avoid equating temporal association with receptor-level causality.

### 6.1. Evidence Hierarchy and Falsifiable Predictions

The convergence model contains three evidence levels. The first is established physiology: GLP-1RAs reduce energy intake, body weight, adipose lipolysis, glycemic exposure, and systemic inflammation. The second is supported intrahepatic biology: semaglutide can engage sinusoidal endothelial GLP-1 receptors in experimental metabolic liver disease, while hepatocyte TAZ responds to cholesterol, cytoskeletal tension, and matrix-derived mechanical signals [8,12,20]. The third remains hypothetical: that either systemic unloading or endothelial signaling is necessary and sufficient to suppress hepatocyte TAZ-TEAD activity. Maintaining this hierarchy prevents association from being presented as mediation.

A causal model predicts that effective GLP-1RA treatment should reduce nuclear hepatocyte TAZ and its transcriptional outputs after controlling for caloric intake and weight change; that loss or constitutive activation of hepatocyte TAZ should respectively attenuate or blunt the antifibrotic response; and that endothelial GLP-1 receptor deletion should modify TAZ-pathway readouts if sinusoidal signaling lies upstream. Failure of these predictions would favor TAZ as a parallel biomarker of improving tissue stress rather than a required mediator.

### 6.2. Multicellular Sequence from Metabolic Unloading to Matrix Remodeling

At the organism level, reduced food intake and weight loss lower the delivery of nonesterified fatty acids from adipose tissue and reduce insulin-driven de novo lipogenesis. Within the liver, this shifts hepatocytes away from lipid oversupply, mitochondrial stress, endoplasmic-reticulum stress, and release of damage-associated signals. These changes can reduce the recruitment and activation of macrophages and lessen the cytokine environment that maintains stellate-cell activation. Improved glycemic control may add benefit by reducing substrate flux and glucotoxic stress, although these effects cannot be separated completely from weight loss in most clinical datasets.

The sinusoidal endothelium provides a second physiological entry point. Healthy liver sinusoidal endothelial cells maintain fenestration, regulate leukocyte trafficking, clear circulating macromolecules, and communicate with hepatocytes, Kupffer cells, and stellate cells. During metabolic liver injury, endothelial dysfunction and capillarization disturb this homeostatic niche. GLP-1 receptor-dependent improvement in endothelial signaling could therefore reduce inflammatory recruitment and indirectly change the mechanical and paracrine environment experienced by hepatocytes and stellate cells [8,14]. This route is compatible with a direct hepatic action of semaglutide without requiring functional GLP-1 receptors on either hepatocytes or stellate cells.

TAZ occupies a downstream position at which several of these inputs may be integrated. Reduced free-cholesterol stress may restore proteasomal turnover of TAZ; improved energy balance may reinforce Hippo-kinase restraint; and slower matrix accumulation may reduce stiffness-dependent nuclear localization. In turn, lower TAZ-TEAD activity would be expected to decrease IHH and other hepatocyte-derived signals that sustain fibrogenic stellate-cell programs. This creates a potential feed-forward interruption: metabolic unloading reduces injury, reduced injury limits TAZ activation, and weaker TAZ-dependent paracrine signaling slows matrix deposition and mechanical reinforcement. Each step is individually plausible, but the complete sequence has not been demonstrated in one receptor-dependent experiment.

### 6.3. Temporal Predictions and Competing Causal Models

A causal convergence model makes temporal predictions. Improvements in appetite, glycemia, and circulating substrates should precede changes in hepatic lipid stress; reductions in hepatocyte injury and inflammatory recruitment should follow; and changes in matrix organization and stiffness should emerge later. If hepatocyte TAZ is a mediator, its nuclear activity and TEAD-dependent transcription should decline before, or at least alongside, reductions in IHH and stellate-cell activation. If TAZ changes only after histological improvement is established, it is more likely to be a downstream marker of tissue recovery than a principal mediator.

At least three competing causal models should be tested. In a systemic-unloading model, reduced energy intake and adipose lipolysis are sufficient to lower hepatocyte TAZ-related stress. In an endothelial model, LSEC GLP-1 receptor activation contributes additional weight-loss-independent signals that modify the inflammatory and reparative niche. In a parallel-response model, GLP-1RA therapy and TAZ activity change independently because both respond to a third process, such as reduced lipotoxicity. Weight-matched pair feeding, endothelial receptor deletion, hepatocyte TAZ rescue, and longitudinal sampling are required to distinguish these alternatives [8,20,22,23].

**Figure 1:**
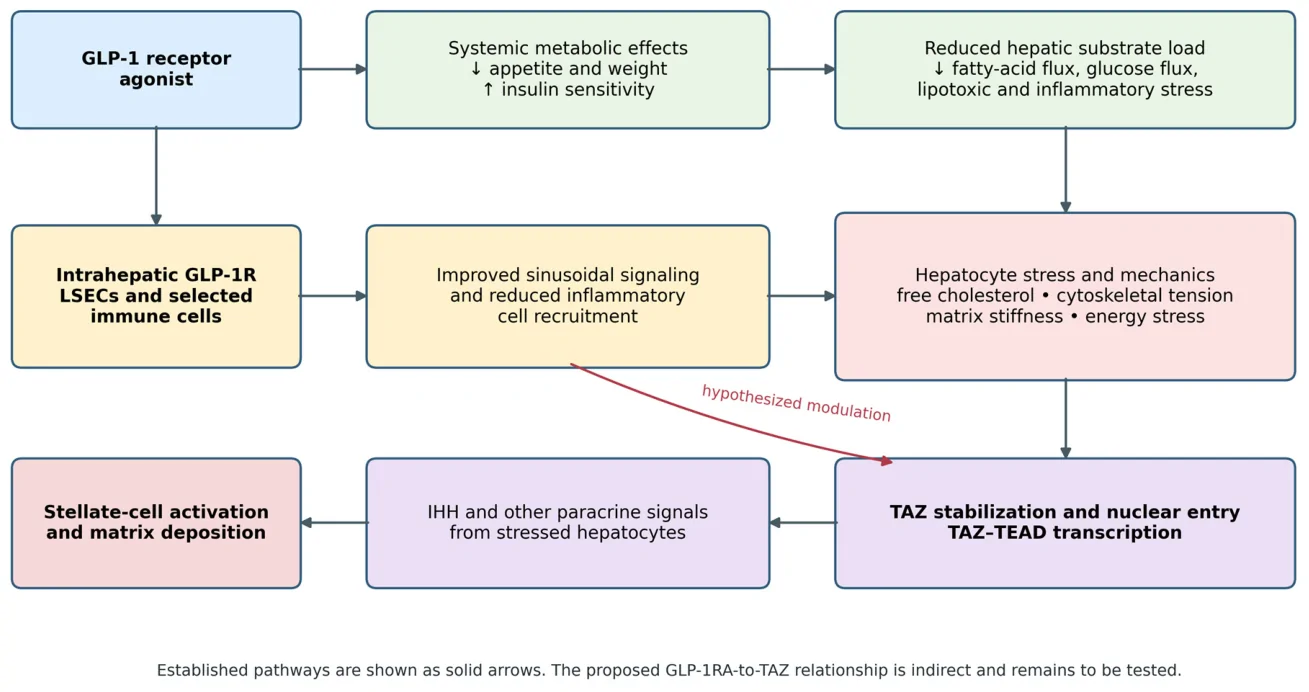
Cell-specific physiological convergence of GLP-1 receptor agonism and TAZ signaling in metabolic liver disease. Solid arrows denote established or experimentally supported pathways. The dashed arrow denotes the proposed, unproven modulation of hepatocyte TAZ by systemic or intrahepatic GLP-1 receptor signaling. GLP-1R, glucagon-like peptide-1 receptor; IHH, Indian hedgehog; LSEC, liver sinusoidal endothelial cell; TAZ, transcriptional coactivator with PDZ-binding motif; TEAD, TEA domain transcription factor.

These models need not be mutually exclusive. Systemic metabolic unloading may dominate early, endothelial signaling may modify local inflammatory traffic and repair, and declining matrix stiffness may reinforce later reductions in mechanosensitive TAZ activity. The relative contribution of each route may also vary by disease stage. Early MASH with limited fibrosis may respond mainly through substrate and inflammatory changes, whereas advanced fibrosis may be constrained by persistent matrix mechanics and slower scar turnover. A stage-specific model is more physiologically credible than a single invariant pathway. The proposed convergence model is illustrated in Figure 1.

## 7. Clinical Evidence Through a Mechanistic Lens

### 7.1. Liraglutide and Early Proof of Concept

The LEAN phase 2 trial established early histological proof of concept for an incretin-based approach in steatohepatitis: liraglutide increased resolution of steatohepatitis and reduced progression of fibrosis compared with placebo [25]. The small sample and secondary nature of fibrosis outcomes limited mechanistic inference, but the study showed that changing systemic metabolic physiology could alter hepatic histology.

### 7.2. Semaglutide: From MASH Resolution to Fibrosis Improvement

In a phase 2 trial, daily semaglutide produced dose-dependent NASH resolution without worsening fibrosis, reaching 59% with 0.4 mg compared with 17% with placebo; however, the prespecified fibrosis-improvement endpoint was not significantly different [5]. This dissociation suggested that inflammatory-metabolic activity may respond faster than established matrix architecture.

The phase 3 ESSENCE interim analysis materially advanced the field. Among patients with biopsy-defined MASH and F2-F3 fibrosis, weekly semaglutide 2.4 mg achieved MASH resolution without worsening fibrosis in 62.9% versus 34.3% with placebo and at least one-stage fibrosis reduction without worsening MASH in 36.8% versus 22.4%, respectively [6,26]. Mean weight change was −10.5% with semaglutide and −2.0% with placebo. These data establish histological efficacy but do not determine whether TAZ activity changes, whether such a change precedes fibrosis improvement, or how much benefit is mediated by weight loss.

### 7.3. Tirzepatide and Multi-Agonist Physiology

The SYNERGY-NASH phase 2 trial tested tirzepatide, a dual GIP/GLP-1 receptor agonist, in biopsy-confirmed MASH with F2-F3 fibrosis. MASH resolution without worsening fibrosis occurred in 44%, 56%, and 62% at 5, 10, and 15 mg, respectively, versus 10% with placebo. At least one-stage fibrosis improvement without worsening MASH occurred in approximately half of tirzepatide-treated participants versus 30% with placebo [16]. Because tirzepatide engages two incretin receptors and produces substantial weight loss, these findings reinforce the importance of metabolic unloading but cannot isolate GLP-1 receptor signaling or TAZ mediation. The principal histology-based incretin trials are summarized in Table 3.

### 7.4. Interpreting Histological Benefit Without Overassigning Mechanism

Histological improvement in the liraglutide, semaglutide, and tirzepatide trials establishes therapeutic efficacy but cannot by itself identify the responsible cell type or molecular mediator. Resolution of MASH may follow reduced nutrient delivery and inflammatory injury, whereas collagen remodeling additionally depends on matrix turnover, stellate-cell state, and time. The stronger fibrosis signal in the phase 3 semaglutide analysis is compatible with sustained upstream unloading, a direct intrahepatic contribution, or both. Because trial biopsies were not designed around TAZ-pathway pharmacodynamics, these alternatives remain unresolved.

Future trials should pair histology with spatially resolved measurements of GLP-1 receptor-positive sinusoidal endothelial cells, hepatocyte nuclear TAZ, TEAD-regulated transcripts, IHH, collagen architecture, and markers of stellate-cell activation. Mediation analysis should incorporate longitudinal weight change, glycemic improvement, and adipose-tissue biomarkers. Such designs could determine whether TAZ reduction precedes fibrosis improvement, simply tracks reduced injury, or identifies a mechanistically distinct responder subgroup.

### 7.5. What Clinical Trials Can and Cannot Resolve

Current trials answer the clinically important question of whether incretin-based therapy improves MASH histology, but they were not designed to identify a TAZ-dependent mechanism. Liver biopsies sample a small and spatially heterogeneous fraction of the organ, are obtained at limited time points, and generally lack validated receptor localization, nuclear TAZ quantification, or TEAD-target measurements. The observed association between weight loss and histological response can support metabolic mediation, but it cannot exclude weight-loss-independent endothelial effects or establish molecular causality [5,6,16,26].

Fibrosis endpoints also require careful interpretation. A one-stage histological improvement integrates collagen quantity, architecture, sampling variation, and reader assessment. It does not directly measure matrix stiffness, cross-linking, collagen turnover, or the activity of stellate-cell subpopulations. Conversely, absence of a statistically significant fibrosis endpoint over 52–72 weeks does not prove absence of an antifibrotic mechanism, because inflammatory resolution and reduced fibrogenic drive may precede visible scar remodeling. Mechanism-rich studies should therefore combine histology with elastography, matrix-turnover markers, digital collagen quantification, and cell-specific molecular assays.

Clinical mediation analysis would be strengthened by repeated measurements rather than baseline and end-of-treatment comparisons alone. Longitudinal body composition, glycemia, circulating fatty acids, liver fat, stiffness, IHH, and other candidate pathway markers could clarify temporal ordering. Prespecified analyses should test whether a TAZ-related biomarker adds explanatory value after accounting for weight loss and metabolic improvement. Such analyses would still be observational within a trial unless paired with an intervention that specifically perturbs the proposed mediator.

## 8. Translational Opportunities

### 8.1. TAZ and IHH as Mechanism-Based Biomarkers

Ideally, a biomarker should reflect target engagement rather than simply reproduce a general signal of liver injury. Circulating IHH is an investigational mechanistic biomarker because it sits downstream of hepatocyte TAZ and is elevated in experimental and human steatohepatitis with fibrosis [24]. Paired measurements of circulating IHH, hepatocyte nuclear TAZ, liver stiffness, extracellular-matrix turnover markers, and histological response could test the hypothesis that incretin therapy engages the TAZ-IHH axis. IHH is not currently a validated clinical biomarker for treatment selection or response monitoring. Such studies should establish assay reproducibility, tissue specificity, temporal ordering, and incremental value beyond FIB-4, elastography, enhanced liver fibrosis scores, and conventional aminotransferases [12,24].

### 8.2. Combination Therapy

No single molecular target is likely to control MASH optimally in every patient. GLP-1RAs address upstream metabolic load and provide cardiovascular and weight-related benefits, whereas a TAZ-TEAD- or IHH-directed intervention could more directly interrupt the hepatocyte-to-stellate-cell fibrogenic signal. This generates a preclinical, hypothesis-driven combination strategy: metabolic unloading plus blockade of maladaptive mechanotransduction. Clinical benefit has not been demonstrated for this combination concept, and safety remains a central challenge. YAP/TAZ participate in tissue repair, regeneration, and organ homeostasis, so cell-selective delivery, partial modulation, or downstream pathway targeting may be preferable to indiscriminate systemic inhibition [6,15,23].

### 8.3. Precision Physiology

The TAZ model may be most relevant in biologically defined subgroups. Hepatic free-cholesterol accumulation, advanced matrix stiffness, and elevated IHH could identify patients whose fibrosis is strongly coupled to TAZ signaling. Conversely, patients dominated by other drivers may improve through TAZ-independent routes. Future trials should therefore integrate lipidomics, spatial cell biology, and noninvasive physiology rather than assuming a uniform mechanism [20,24]. The causal questions and experiments required to test this model are summarized in Table 4.

### 8.4. Translational Constraints and Safety Considerations

Translation of TAZ-directed therapy requires caution because YAP and TAZ contribute to physiological regeneration, epithelial integrity, vascular biology, and tissue repair. Broad, sustained systemic inhibition could therefore impair adaptive responses outside the liver or interfere with recovery from acute injury. The therapeutic objective in MASH may be partial, cell-selective, or time-limited modulation rather than complete pathway suppression. Hepatocyte-targeted nucleic-acid delivery, disruption of a disease-specific downstream signal, or treatment during a defined window of active fibrogenesis may provide a wider safety margin than systemic TEAD inhibition [11,15,22,23].

Combination strategies must also demonstrate more than additive changes in biomarkers. A rational combination would pair complementary mechanisms, such as upstream metabolic unloading with selective interruption of hepatocyte-to-stellate-cell signaling, while avoiding overlapping adverse effects. Preclinical factorial designs should compare each monotherapy with the combination, include exposure-matched controls, and evaluate regeneration, wound healing, tumor surveillance, and extrahepatic toxicity. Without these controls, improved fibrosis could reflect greater overall treatment intensity rather than true mechanistic complementarity.

A clinically useful precision strategy would require a reproducible marker that identifies patients with active TAZ-IHH signaling and predicts incremental benefit from pathway modulation. Circulating IHH is promising as a mechanistic research marker, but its tissue specificity, analytical performance, biological variability, and responsiveness to treatment remain insufficiently characterized for clinical selection [24]. Biomarker development should therefore proceed in parallel with assay validation and tissue-based confirmation rather than assuming that a circulating concentration uniquely represents hepatocyte TAZ activity.

## 9. Research Priorities

First, the principal translational question is whether the genetically supported LSEC GLP-1R mechanism identified in mouse MASH is conserved in human disease. Human studies should use validated, orthogonal, and spatially resolved methods to examine GLP1R expression and function in sinusoidal endothelial and immune subpopulations across disease stages, while continuing to test rather than assume receptor activity in hepatocytes and stellate cells. Second, studies should use clinically relevant exposures and include pair-fed or weight-matched controls to distinguish pharmacological signaling from reduced energy intake. Third, TAZ should be assessed as activity rather than expression alone: nuclear localization, phosphorylation, TEAD occupancy, and downstream transcription are more informative than total protein. Fourth, co-culture, organoid, precision-cut liver slice, and humanized-liver systems should preserve intercellular signaling and matrix mechanics. Fifth, clinical trials should collect longitudinal biospecimens early enough to determine whether changes in the LSEC–hepatocyte–stellate-cell—axis, nuclear TAZ, TAZ-IHH signaling, and fibrosis architecture precede histological improvement. Finally, causal mediation analyses should integrate weight change, insulin sensitivity, liver fat, inflammatory markers, cell-specific GLP-1R readouts, TAZ activity, and fibrosis outcomes.

One practical translational design would pair an in vitro physiology study with a clinical biomarker analysis. Hepatocytes exposed to palmitate/oleate and cholesterol could be treated with semaglutide under receptor-verified conditions, followed by conditioned-medium transfer to stellate cells. Parallel patient samples obtained before and during GLP-1RA therapy could test whether the same TAZ-IHH signature falls in responders. Concordance across these levels would provide a stronger causal chain than either cell culture or clinical association alone.

## 10. Limitations of the Current Evidence

The proposed connection is supported by strong, but still largely parallel, lines of evidence: TAZ is mechanistically implicated in experimental steatohepatitis [12,20,21,22,23], and incretin therapies improve clinical and histological outcomes [5,6,16,25], but direct intervention studies linking GLP-1R signaling to TAZ regulation are absent. Animal models incompletely reproduce human MASH, cell lines may not express receptors or mechanical environments faithfully, and weight loss creates pervasive confounding [1,10,19]. TAZ and YAP are often measured together despite distinct functions, and whole-liver assays obscure cellular origin [10,19]. Trial biopsies provide limited spatial and temporal resolution [5,6,16]. Finally, MASH nomenclature and treatment standards are evolving, requiring care when comparing studies conducted under earlier NAFLD/NASH definitions [1].

## 11. Conclusions

GLP-1 receptor agonism and TAZ biology intersect at a fundamental physiological problem: how systemic nutrient excess is converted into cellular stress, maladaptive intercellular communication, and fibrotic tissue mechanics. GLP-1RAs reduce upstream metabolic burden and improve steatohepatitis and fibrosis in appropriately selected patients. Recent evidence for functional GLP-1 receptors in liver sinusoidal endothelial cells adds a direct intrahepatic, weight-loss-independent mechanism to the established systemic model. In parallel, hepatocyte TAZ translates cholesterol, cytoskeletal tension, and matrix stress into profibrotic paracrine signals, particularly IHH. These independently supported pathways can potentially converge through endothelial–hepatocyte–stellate-cell—communication, but TAZ has not been established as a downstream mediator of GLP-1 receptor agonism. The next advance requires receptor-validated, cell-specific, weight-controlled experiments and longitudinal human biomarker studies capable of distinguishing causal mediation from parallel improvement. This evidence-based boundary defines the proposed GLP-1RA-TAZ relationship as a precise experimental framework rather than a presumed mechanism.

## Figures and Tables

**Table 1:** Evidence for GLP-1 receptor expression and function in major hepatic cell populations.

Cell Population	Evidence	Assessment Approach	Current Interpretation
Hepatocytes	Positive transcript/protein and ligand-response findings were reported in primary human hepatocytes and hepatoma-derived lines [17]. In contrast, validated tissue localization and later functional studies did not establish meaningful hepatocyte GLP-1R signaling [7,13].	RT-PCR, immunodetection, ligand internalization and metabolic assays; validated monoclonal-antibody localization; receptor profiling and functional agonist testing.	Conflicting evidence; direct hepatocyte GLP-1R signaling is not established.
Hepatic stellate cells	No meaningful direct response to GLP-1 or GIP receptor agonism was detected in human hepatic stellate-cell preparations [7].	Receptor profiling and functional agonist testing.	Direct stellate-cell signaling is not supported by current human-cell evidence.
Liver sinusoidal endothelial cells	Glp1r was localized predominantly to pericentral LSECs in mouse MASH. Endothelial deletion or knockdown attenuated semaglutide-mediated hepatic benefit despite preserved weight loss [8].	Cell-resolved profiling, Glp1rTie2-/- mice, and AAV8-Cre-mediated hepatic endothelial knockdown.	Strong cell-specific and genetic evidence in mouse MASH; conservation in human MASH is unresolved.
CD8+ T cells	Intrahepatic Glp1r expression was detected in CD8+ T-cell populations in experimental MASH [8].	Cell-resolved expression analysis.	Expression is supported in mice, but its independent contribution to hepatic treatment response requires clarification.
Kupffer cells and other macrophages	Current evidence does not establish a receptor-dependent direct response sufficient to explain GLP-1RA benefit.	Cell-resolved receptor profiling and functional inference.	Effects are more plausibly secondary to systemic or endothelial signaling until receptor dependence is demonstrated.

**Table 2:** Physiological levels linking GLP-1 receptor agonism to hepatic remodeling.

Level	Established or Plausible Effect	Potential Relationship to TAZ
Gut–brain axis	Reduced appetite, delayed gastric emptying, and lower energy intake [2,3,4]	Lower nutrient overload may indirectly reduce hepatocyte stress signals that favor TAZ stabilization.
Pancreatic islet	Glucose-dependent insulin secretion and context-dependent glucagon suppression [2,3,4]	Improved insulin-to-glucagon physiology may reduce hepatic glucose and lipid flux; direct TAZ mediation is unproven.
Adipose tissue	Weight loss, improved insulin sensitivity, and reduced lipolytic fatty-acid delivery [2,3,5,6]	Reduced fatty-acid and cytokine exposure may decrease lipotoxic and cytoskeletal stress upstream of TAZ.
Hepatocyte	Reduced steatosis and inflammatory injury after effective incretin therapy; changes in free-cholesterol stress remain mechanistic hypotheses [5,6,7,20]	These changes may reduce nuclear TAZ and TAZ-TEAD targets such as IHH; direct GLP-1 receptor dependence remains uncertain.
Immune compartment	Reduced inflammatory activity is biologically plausible and supported by clinical and experimental evidence [5,8,10]	Lower cytokine amplification may interrupt YAP/TAZ-associated inflammatory circuits.
Stellate cell and matrix	Reduced injury signaling may eventually lower matrix production and stiffness [9,14,15,20]	Reduced stiffness may decrease integrin-Rho-actin-driven TAZ activation, weakening a feed-forward loop.

**Table 3:** Selected histology-based incretin trials in steatohepatitis.

Study	Population and Intervention	Key Histological Finding	Mechanistic Interpretation
LEAN [25]	52 adults; liraglutide 1.8 mg daily; 48 weeks	Higher steatohepatitis resolution and less fibrosis progression versus placebo.	Proof of concept; too small to define molecular mediation.
Phase 2 semaglutide [5]	320 adults with F1-F3; daily semaglutide; 72 weeks	NASH resolution 59% at 0.4 mg vs. 17% placebo; fibrosis improvement not significantly different.	Inflammatory-metabolic response may precede matrix regression.
SYNERGY-NASH [16]	190 adults with F2-F3; tirzepatide 5–15 mg weekly; 52 weeks	MASH resolution 44–62% vs. 10%; fibrosis improvement 51–55% vs. 30%.	Supports metabolic unloading; dual agonism prevents attribution to GLP-1 alone.
ESSENCE interim [6,26]	800-participant interim cohort with F2-F3; semaglutide 2.4 mg weekly; 72 weeks	MASH resolution 62.9% vs. 34.3%; fibrosis improvement 36.8% vs. 22.4%.	Confirms histological efficacy; mechanism-rich samples are needed to test TAZ mediation.

**Table 4:** Proposed evidence chain and experiments needed to establish causality.

Question	Minimum Experiment	Decisive Readouts
Does GLP-1RA exposure alter TAZ?	Dose- and time-course studies in metabolically stressed primary human hepatocytes or well-characterized hepatic models.	Total and nuclear TAZ; p-TAZ/TAZ ratio; WWTR1 abundance; TEAD reporter activity; IHH and CTGF/CYR61 expression [12,20,21,22,23].
Is the effect receptor dependent?	Confirm GLP1R with validated transcript/protein methods, then use receptor knockout/knockdown and a validated antagonist.	Loss of drug effect after receptor disruption, with preserved response to downstream cAMP controls.
Is the effect secondary to lipid unloading?	Match intracellular lipid/cholesterol reduction across pharmacological and nutritional conditions.	TAZ change after controlling for triglyceride, free cholesterol, oxidative stress, and cell viability.
Which liver cell mediates the response?	Cell-specific cultures and hepatocyte–stellate–cell/macrophage co-cultures.	Cell-resolved TAZ localization, secretome profiling, stellate-cell ACTA2/COL1A1, collagen deposition.
Does the pathway operate in humans?	Paired liver tissue or mechanism-rich biopsies from treated MASH cohorts.	Nuclear hepatocyte TAZ, IHH, spatial transcriptomics, fibrosis markers, and association with histologic response [6,8,22,26].
Is TAZ a mediator rather than a marker?	TAZ rescue or constitutive activation during GLP-1RA treatment; factorial GLP-1RA plus TAZ/TEAD inhibition.	Attenuation or enhancement of antifibrotic benefit and formal mediation analysis.

## Data Availability

No new data were created or analyzed in this study. Data sharing is not applicable to this article.

## Abbreviations

The following abbreviations are used in this manuscript:

GLP-1	Glucagon-like peptide-1
GLP-1RA	Glucagon-like peptide-1 receptor agonist
IHH	Indian hedgehog
MASLD	Metabolic dysfunction-associated steatotic liver disease
MASH	Metabolic dysfunction-associated steatohepatitis
TAZ	Transcriptional coactivator with PDZ-binding motif
TEAD	TEA domain transcription factor
WWTR1	WW domain-containing transcription regulator 1
